# Arrhythmia Detection by Data Fusion of ECG Scalograms and Phasograms

**DOI:** 10.3390/s24248043

**Published:** 2024-12-17

**Authors:** Michele Scarpiniti

**Affiliations:** Department of Information Engineering, Electronics and Telecommunications (DIET), Sapienza University of Rome, Via Eudossiana 18, 00184 Rome, Italy; michele.scarpiniti@uniroma1.it; Tel.: +39-06-44585869

**Keywords:** arrhythmia, ECG classification, continuous wavelet transform (CWT), scalogram, phasogram, deep learning

## Abstract

The automatic detection of arrhythmia is of primary importance due to the huge number of victims caused worldwide by cardiovascular diseases. To this aim, several deep learning approaches have been recently proposed to automatically classify heartbeats in a small number of classes. Most of these approaches use convolutional neural networks (CNNs), exploiting some bi-dimensional representation of the ECG signal, such as spectrograms, scalograms, or similar. However, by adopting such representations, state-of-the-art approaches usually rely on the magnitude information alone, while the important phase information is often neglected. Motivated by these considerations, the focus of this paper is aimed at investigating the effect of fusing the magnitude and phase of the continuous wavelet transform (CWT), known as the scalogram and phasogram, respectively. Scalograms and phasograms are fused in a simple CNN-based architecture by using several fusion strategies, which fuse the information in the input layer, some intermediate layers, or in the output layer. Numerical results evaluated on the PhysioNet MIT-BIH Arrhythmia database show the effectiveness of the proposed ideas. Although a simple architecture is used, their competitiveness is high compared to other state-of-the-art approaches, by obtaining an overall accuracy of about 98.5% and sensitivity and specificity of 98.5% and 95.6%, respectively.

## 1. Introduction

One of the main causes of death worldwide is due to cardiovascular diseases [1,2]. In fact, the World Health Organization in 2021 underlined 32% of all global deaths as being due to cardiovascular diseases [3]. Cardiac arrhythmia, which pertains to any irregular change from normal heart rhythms, is a common cardiovascular disease [4]. Arrhythmia can be detected by electrocardiogram (ECG), a simple and non-invasive tool for analyzing the rhythm and status of the heart. In addition, ECG is well known to contain much information on heart diseases. For this purpose, the development of automatic approaches for the detection and classification of cardiac arrhythmia by analyzing the ECG signal is very important [5].

Detection of arrhythmia from ECG signals has been deeply investigated from many years using different families of approaches. For example, past attempts are based on signal processing techniques and adaptive filtering [6], such as Kalman filters [7] or decision rules [8]. However, most past approaches were based on extracting a suitable set of features (such as ECG morphology and heartbeat intervals) and exploiting a specific type of classifier, usually based on neural networks (NNs) [9] or support vector machines (SVMs) [10].

Although these machine learning approaches provide reasonable results in terms of sensitivity and specificity, and SVMs are still employed as a classifier [11], the recent advent of deep learning techniques [12] has generated new research lines in the field [13,14] and produced a high speedup in recent years [15]. The main techniques are based on the use of convolutional neural networks (CNNs) [16,17], recurrent neural networks (RNNs) [18,19], or their combination [20,21]. Among all approaches, CNNs are the most used and preferable one due to their performance and computational efficiency. In literature, both 1D CNNs [22] and 2D CNNs [23] working directly on the ECG signal in an end-to-end fashion are used. However, a plethora of approaches exploit an image-like bi-dimensional representation of the ECG signal [13,14] by transforming the ECG in a suitable image [24].

Among these bi-dimensional CNN-based approaches, some works focused on the use of the wavelet transform [17,25,26,27]. However, instead of using the (discrete) wavelet transform of the signal for denoising and feature extraction, many authors propose exploiting the squared magnitude of the continuous wavelet transform (CWT), called the *scalogram* [28,29,30,31,32]. Scalograms, similarly to spectrograms, are a bi-dimensional representation of signals involving the two-dimensional time-scale domain. Moreover, scalograms, differently from spectrograms, overcome the trade-off between the time and frequency resolution typical of the STFT, providing a non-constant resolution at different time-scales.

Different families of wavelet functions have been used in literature, involving both real-valued and complex-valued (or analytic) wavelets. However, analytic wavelets have some advantages over the real ones since they show a superior time localization. In addition, the use of a complex-valued wavelet allows us to separate the phase and magnitude information of signals in the analysis of the time evolution of frequency components [33]. In this work, we adopt the complex Morlet wavelet that provides very good time–frequency localized information and is largely used for biological signal analysis [34]. Similarly to the scalogram, in the case of using a complex-valued wavelet family, it is possible to also provide a bi-dimensional image-like representation of the phase information, called the *phasogram*. Specifically, magnitude and phase, although strongly related, do not properly carry the same information; however, they do have complementary information and the exploitation of both magnitude and phase often provides advantages and improvements in performance. This kind of approach is very common, for example, in speech processing. In fact, in this field, a frequent practice relies on the use of complex spectrogram, i.e., the complete STFT, as investigated in [35] for speech enhancement and in [36] for speech generation.

However, instead of working with a complex-valued representation, many authors prefer to work separately with magnitude and phase, and fuse this information, like in works [37,38,39]. Recently, this idea is also exploited in other fields, such as action recognition in Wi-Fi signals using amplitude and phase information from CSI (Channel State Information) [40], the detection of defects in images using non-destructive techniques [41], and the augmentation of sounds related to construction site equipment [42].

**Figure 1:**
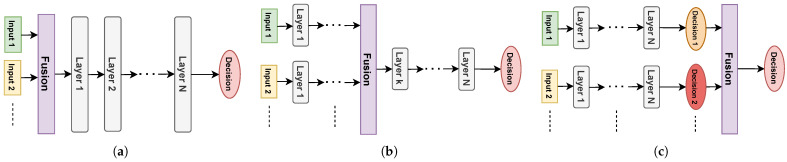
Schemes of the early, intermediate, and late fusion strategies. (**a**) Early fusion. (**b**) Intermediate fusion. (**c**) Late fusion.

Since scalograms and phasograms are bi-dimensional representations, like gray-scale images, they are suitable to be used as input to CNNs that perform well on image data types. Moreover, due to the complementarity of the magnitude (scalogram) and phase (phasogram) information, scalograms and phasograms can be used in deep learning architectures by exploiting data fusion (DF) [43]. To this aim, there exist different fusion strategies according to the model layer in which the fusion is performed. Specifically, we distinguish them as early, intermediate, and late fusion strategies, where the fusion is made at the input, in an intermediate layer, and at the output, respectively, as shown in Figure 1. In early fusion, raw input data from different sources are merged into a single vector, which is processed as a unique input (see Figure 1a). Although a simple approach, early fusion enables learning of cross-interactions from the different inputs. However, it can struggle to capture relationships between inputs that emerge only at higher levels of abstraction since it does not explicitly model individual input-specific representations. Intermediate fusion takes a different approach by first learning input-specific feature representations and then combining them in an intermediate layer of the network, rather than merging the raw data (see Figure 1b). This strategy can more accurately capture complex cross-interactions and often yields more meaningful representations. Finally, in late fusion, rather than combining raw data or intermediate features, decisions are made by separate models and then integrated into a final decision (see Figure 1c). This approach ensures that each model can focus on optimizing its performance for its specific input. Additionally, since the errors made by single models may be uncorrelated, they can complement each other. However, late fusion has a key limitation since it cannot capture cross-interactions at the data or feature levels, as these are not explicitly modeled by the final decision layer.

Motivated by these considerations, in this paper, we aim at investigating the effect of exploiting both the scalograms and phasograms of ECG signals by implementing different early, intermediate, and late fusion strategies to identify the most effective one for the detection of arrhythmia. Since the focus of the paper is not on introducing novel architectures but, instead, on the evaluation of the simultaneous exploitation of both magnitude and phase information extracted by the CWT and its advantage over using the scalogram alone, simulations are performed by using a simple CNN model, i.e., AlexNet [44]. The use of such a network, composed of a short sequence of convolutional layers and presenting a limited computational complexity, makes it easier to evaluate the effect of the fusion of scalograms and phasograms. Experimental validation was performed on the well-known MIT-BIH database [45], which is a highly imbalanced dataset. This database is recommended by the American Association of Medical Instrumentation (AAMI) because it includes five essential arrhythmia groups. Interestingly enough, the proposed solutions, although a simple model is used, have performed well and strongly mitigated the negative effect of data imbalance, being competitive with respect to other state-of-the-art approaches.

The rest of the paper is composed as follows: Section 2 discusses the related work. Section 3 introduces the proposed methodology and the implemented experimental setup, while Section 4 shows the obtained numerical results and related discussion. Finally, Section 5 concludes the work and outlines some future research lines.

## 2. Related Work

The literature about automatic ECG arrhythmia detection and classification is rich and embraces several approaches [46]—from the older ones based on the morphological and rhythm analysis of the recorded waveform [47], adaptive filtering [6], or hidden Markov models [48], to the modern ones based on deep learning techniques [13].

### 2.1. Traditional and Basic Machine Learning Approaches

Traditional approaches were mainly based on signal processing and filtering techniques used to produce a clean ECG signal for detecting peculiar waveform tracts, like the QRS morphology and the RR interval, which for a long time have constituted the major features for computerized arrhythmia monitoring [9,47,49].

More recently, machine learning-based methods have been proposed. Specifically, such approaches are usually based on signal segmentation, manual feature extraction, and the use of classifiers like support vector machines (SVMs), kNN, and random forests [27,50,51]. Often, features are extracted by using higher-order statistics (HOS) [52,53,54]. In addition, the work in [55] exploited the use of the Fourier decomposition for a multi-scale analysis of ECG signal by extracting time-domain and statistical features, and obtaining satisfactory results on the MIT-BIH Arrhythmia dataset. However, the preprocessing and manual feature extraction procedures make these methods of limited applicability in real scenarios in favor of deep learning approaches.

### 2.2. Deep Learning Approaches

About deep learning methods, we mention those based on deep neural networks (DNNs) [56], (stacked) autoencoders [57], deep belief networks (DBNs) [58,59], recurrent neural networks (RNNs) [60], both 1D [22] and 2D [23,61,62] convolutional neural networks (CNNs), U-Nets [63,64], extreme learning machines (ELMs) [19,25], capsule networks [65], transformer [66], and hybrid models [29].

Specifically, the work proposed in [56] introduced a simple DNN working on a filtered version of the ECG signal after a suitable segmentation phase. In [57], Luo et al. proposed an automatic feature extraction mechanism based on a stacked-autoencoder (AE) working on the wavelet transform of the EGC followed by a DNN classifier. The work in [22] showed the effectiveness of restricted Boltzmann machines and deep belief networks in arrhythmia detection, even if accuracy never exceeds 95%. Warrick et al. in [60] constructed a recurrent neural network based on bi-directional LSTM units working on the time scattering transform and phase-harmonic correlation coefficients extracted from each ECG channel. The use of extreme learning machines was investigated in [19], which proposed extracting features by using a gated recurrent neural network, and in [25], which exploited CNNs as the feature extractor. An end-to-end approach based on U-Nets was proposed in [63] and also in [64], which proposed a modified version implementing the Winograd convolution. The recent work in [66] investigated the use of transformers in different learning paradigms for improving the model efficiency. Good results were obtained by a transformer-based CNN architecture in [67]. Also, a convolution-free transformer-based approach was proposed in [68] but the results were not very enthusiastic.

#### 2.2.1. The Use of 1D CNNs

Although there exist several approaches in arrhythmia detection, the main literature is focused on the use of 1D and 2D CNNs for extracting meaningful features from the ECG signal, also exploiting transfer learning (TL) [69,70]. Specifically, the use of 1D CNNs is quite common to analyze time-series and provided good results also in arrhythmia detection [22,71,72,73,74]. Similarly, ref. [75] exploited a 1D CNN to extract features used then in an LSTM network, while authors in [17] investigated the use of a ResNet-18 architecture. Moreover, the work in [76] proposed equipping the 1D CNN with an additional non-local attention module to better capture long-range dependencies. The approach proposed in [77] is based on a domain-adaptive classification exploiting both CNNs and an unsupervised domain adaptation that uses a specific loss function based on the observation of clustering characteristics.

There are also some hybrid approaches that combine 1D CNN and LSTM [29,78,79] or DenseNet and Bi-directional LSTM [80]. For example, Jiao et al. in [65] proposed a capsule network-based ECG classification approach implementing an LSTM network and a 1D CNN as a parallel feature extraction layer to extract the spatial and temporal features of the ECG signal. Finally, the authors of [81] proposed a combination of BiGRU-BiLSTM and a multilayered dilated CNN to detect arrhythmia. This kind of approach generally performs quite well in detecting arrhythmia. However, performance can be improved by using different and richer representations of the ECG signals.

#### 2.2.2. The Use of 2D CNNs

Since CNNs have been demonstrated to be very powerful in image analysis, the majority of recent approaches are based on the 2D version of the CNN. Some approaches, such as those in [23,82], applied 2D CNN directly to the ECG waveform image. Despite their simplicity, these approaches showed some limitation in performance due to the fact that it is difficult to capture meaningful features from a “picture” of the ECG waveform, particularly if some background noise is present. To overcome this issue, many works exploited a bi-dimensional representation of the ECG signal [24]. To this purpose, Zhai and Tin used a dual-beat coupling matrix [83] as representation. Many other authors instead used time–frequency representations, such as Katal et al. who used spectrograms [84,85], which is a powerful representation. Similarly, many works exploited the squared magnitude of the wavelet transform, known as the scalogram. Specifically, scalogram-based approaches were proposed in [28,31,32,84,86,87,88] by implementing deep learning classifiers usually based on CNNs. Scalograms have been demonstrated to be a good representation providing very good results and taking some advantages with respect to spectrograms, as underlined in [30] and in [89]. Although all these works demonstrated the effectiveness of the scalogram-based representation, the effect of the exploitation of the phase information (by means of the phasogram representation, for example) has never been investigated.

### 2.3. Data Fusion (DF)-Based Approaches

In order to further improve results, some authors proposed exploiting an ensemble of deep learning techniques, like Li et al. in [90] and the work in [91] or data fusion (DF) approaches [92,93,94,95,96,97,98,99,100]. Specifically, multi-information fusion was proposed in [92], able to simultaneously capture both morphological and temporal information from the inputs by exploiting a combination of CNNs and LSTMs for enhancing the extracted features. In [93], Lu et al. proposed a combination of features extracted by a CNN with morphological information taken from the PQRST parameters and showed that their approach performed well on imbalanced data. The authors of [94] proposed three fusion strategies working in a wearable IoT device, which fuse RR intervals obtained from a two-lead ECG. Similarly, the work in [95] proposed a late fusion strategy that uses a majority voting approach on the classification obtained by multiple physiological signals. The authors of [96] proposed an early fusion of three image-like representation of the ECG; specifically, they used the Gramian Angular Field (GAF), the Recurrence Plot, and Markov Transition Field, obtaining good results. In [97], Yang et al. proposed a cascaded 1D CNN architecture and an expert feature extractor exploiting the temporal correlation and spatial variability between multiple leads. The work in [98] was focused on the fusion of three subsets of features obtained from the RR intervals, the discrete wavelet transform, and a CNN followed by an SVM classifier. In [99], Han et al. proposed a multi-instance learning paradigm that fuses features extracted by a 1D CNN working on the ECG waveform and a 2D CNN working of the GAF representation with a final softmax classifier. Finally, the work in [100] introduced an approach that fuses features extracted by two lightweight CNN models but is able to obtain good results. However, these works do not consider the phase contribution in the fused information, which, indeed, can positively impact on the final result, and do not analyze where the fusion is performed inside the network.

Regarding the fusion of the magnitude and phase information, which carry complementary information, there are many works in speech processing, like papers [37,38,39] addressing the speech enhancement problem, where it is a well-established methodology derived from the use of complex spectrograms [35,36]. However, more recently, this approach has also been extended to other fields, such as the action recognition exploiting Wi-Fi signals [40], the detection of defects in images using non-destructive techniques [41], and the augmentation of sounds related to construction site equipment [42]. To the best of our knowledge, the fusion of magnitude and phase information, specifically the fusion of scalograms and phasograms, has never been investigated in the ECG analysis and/or arrhythmia detection.

Overall, motivated by the current state of the art, in this paper, we investigate different data fusion strategies of both scalogram and phasogram representations, filling the gap in the ECG literature with respect to other fields. We expect, as shown in Section 4, that the exploitation of both magnitude and phase information may outperforms current approaches usually relying on the magnitude information alone.

## 3. Methodology

In this section, we introduce the proposed methodology in terms of dataset, preprocessing, extracted features (scalograms and phasograms), implemented architectures, data fusion strategies, settings of hyper-parameters, and evaluation metrics.

### 3.1. The Dataset

**Figure 2:**
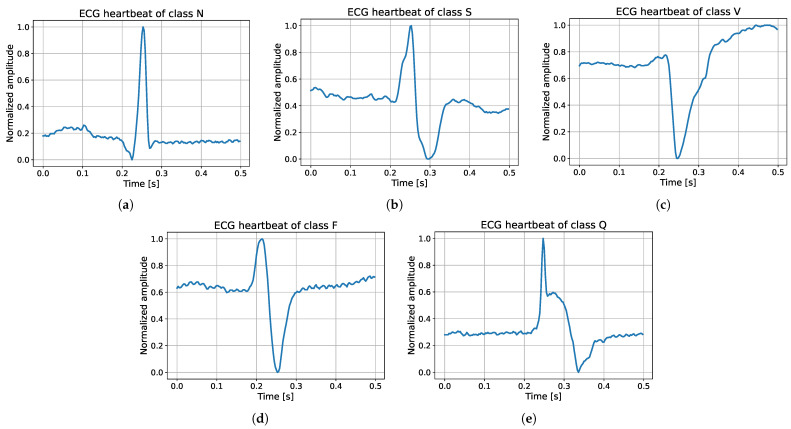
Example of random ECG heartbeats extracted from classes N, S, V, F, and Q, respectively. (**a**) Class N. (**b**) Class S. (**c**) Class V. (**d**) Class F. (**e**) Class Q.

The MIT-BIH Arrhythmia Database [101] is freely available online (it can be downloaded at https://www.physionet.org/content/mitdb/1.0.0/, accessed on September 16, 2024) from the PhysioNet portal [102]. This dataset is quite common in literature since several works show results evaluated on the MIT-BIH Arrhythmia Database [45]: it was estimated that about 60% of works use this dataset for arrhythmia classification [13]. The MIT-BIH Arrhythmia Database is composed of 48 half-hour excerpts of two-channel ambulatory ECG recordings, obtained from 47 subjects. In this work, only the modified lead II was used as input. The recordings were digitized by using a sampling rate of 360 Hz and 11-bit resolution over a 10 mV range. According to the Association for the Advancement of Medical Instrumentation (AAMI), the normal and arrhythmic beats can be grouped into five classes, as shown in Table 1. The dataset is highly imbalanced: in fact, after segmenting it into heartbeats, about 82.77% of the instances are of the normal (N) class, 9.88% are related to three arrhythmia classes (S + V + F; 2.54% for supraventricular ectopic beats (S), 6.61% for premature ventricular contractions (V), and 0.73% for fusion of ventricular and normal beats (F)), and 7.34% are paced or unknown beats (Q). Some normalized heartbeat samples of the five classes are shown in Figure 2. The figure shows segments of half a second centered on the peaks. The dataset contains a total of 109,446 ECG beats and was split into a training set (80%) and a test set (20%), as detailed in Table 1. The imbalance of the MIT-BIH dataset was handled by implementing stratified sampling: the distribution of the target classes among the training and test splits was kept the same, as can be seen analyzing last columns in Table 1. The same proportion among classes between training and test samples usually results in better predictions.

### 3.2. Data Preprocessing

Before extracting the scalograms and phasograms, the raw ECG modified lead II data were preprocessed. Specifically, the following steps were performed [22,51,56]:

- The signal was denoised by applying a band-pass filter between 5 and 20 Hz;
- The filtered signal was resampled to 250 Hz;
- The R-peaks were detected by using the XQRS detector available in the WFDB library shared on the PhysioNet website;
- The ECG signal was segmented into single beats with 180 samples around the identified peaks;
- Each beat was normalized in the 0–1 interval;
- The scalogram and phasogram of each beat was extracted by applying the CWT (see next subsection).

**Figure 3:**
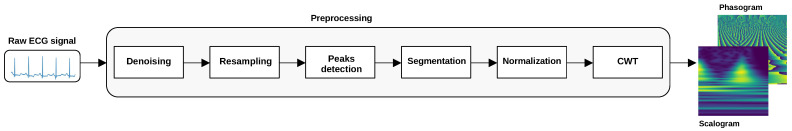
Overview of the preprocessing procedure.

An overview of the whole preprocessing procedure is shown in Figure 3.

### 3.3. Feature Extraction

The use of the continuous wavelet transform (CWT) allows us to overcome the trade-off between the time and frequency resolutions, typical of the STFT [33].

The CWT of a stationary signal x(t) is defined as the product of x(t) with the following basis function family:

(1) $$\Psi_{\tau ,a}\left(t\right)=\frac{1}{\sqrt{a}}\Psi \;\left(\frac{t-\tau }{a}\right),$$

where a>0 is a scaling factor, while τ is the time delay, i.e., $\Psi_{\tau ,a}\left(t\right)$ is a scaled and translated version of the mother waveletfunction $\Psi \;\left(t\right)$. Hence, the CWT of signal x(t) is evaluated as

(2) $$W_{x}\left(\tau ,a\right)=\frac{1}{\sqrt{a}}\int_{-\infty }^{\infty }x\left(t\right)\;\Psi^{*}\;\left(\frac{t-\tau }{a}\right)dt,$$

where ${\,}^{*}$ represents the complex conjugation operator. The delay parameter τ indicates the temporal position of the wavelet $\Psi_{\tau ,a}\left(t\right)$, while the scaling factor *a* determines its frequency characteristics. When 0<a<1, the wavelet $\Psi_{\tau ,a}\left(t\right)$ is primarily focused on high frequencies. Conversely, when a>1, the wavelet $\Psi_{\tau ,a}\left(t\right)$ is concentrated towards lower frequencies. Since the wavelet analysis is performed by varying the *a* and τ parameters, the CWT is particularly effective for analyzing non-stationary signals that include high-frequency transients combined with long-lasting, low-frequency elements [33].

The squared absolute value of the CWT is called the scalogram, and it is defined as

(3) $$S\left(\tau ,a\right)≜{\left|W_{x}\left(\tau ,a\right)\right|}^{2}=\frac{1}{a}{\left|\int_{-\infty }^{\infty }x\left(t\right)\Psi^{*}\;\left(\frac{t-\tau }{a}\right)dt\right|}^{2}.$$

In a similar way, the phase P(τ,a) of the CWT is called the phasogram, and it is defined as

(4) $$P\left(\tau ,a\right)≜∠W_{x}\left(\tau ,a\right)=∠\left(\frac{1}{a}\int_{-\infty }^{\infty }x\left(t\right)\Psi^{*}\;\left(\frac{t-\tau }{a}\right)dt\right).$$

The scalogram S(τ,a) and the phasogram P(τ,a) provide, respectively, a bi-dimensional graphical representation of the signal energy and the signal phase at the specific scale parameter *a* and time location τ.

In general, the mother wavelet Ψ(t) can be any band-pass function and many families have been proposed, both real-valued and complex-valued (Haar, Daubechies, Mexican Hat, the bump wavelet, etc.) [33]. One of the best-performing complex wavelets is the Morlet one, which offers an excellent time resolution and provides very good time–frequency localized information. The Morlet wavelet, a suitable instrument to analyze signals with transients, consists of a complex exponential function multiplied by a Gaussian window and is defined as follows:

(5) $$\Psi \;\left(t\right)=\frac{1}{\sqrt{2\pi \sigma^{2}}}\;e^{-\frac{t^{2}}{2\sigma^{2}}}e^{j\omega_{0}t},$$

where $\omega_{0}$ represents the central frequency of the mother wavelet (the carrier), and $\sigma^{2}$ is the variance of the Gaussian window, given by $\sigma =n/\omega_{0}$. The parameter *n*, referred to as the number of wavelet cycles and set to n=6 in this paper, determines the balance between time and frequency resolutions.

The Morlet wavelet has a huge number of applications in biological signal analysis [34], and its effectiveness as a powerful tool in ECG analysis has been widely demonstrated in several scenarios [103,104].

**Figure 4:**
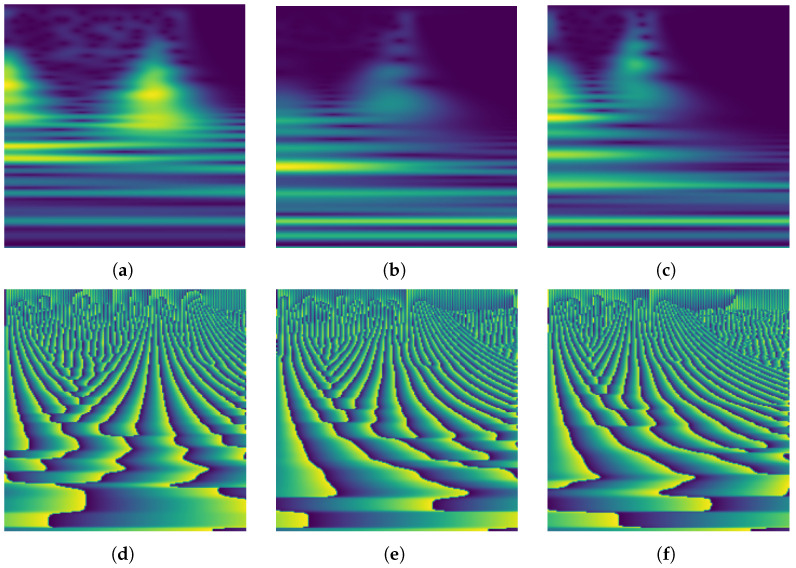
Example of some scalograms (top row) and related phasograms (bottom row) corresponding to three instances extracted from classes N, F, and V, respectively. (**a**) Class N. (**b**) Class V. (**c**) Class F. (**d**) Class N. (**e**) Class V. (**f**) Class F.

Examples of some scalograms and related phasograms corresponding to three random instances extracted from classes N, F, and V, respectively, are shown in Figure 4.

### 3.4. Architecture

**Figure 5:**
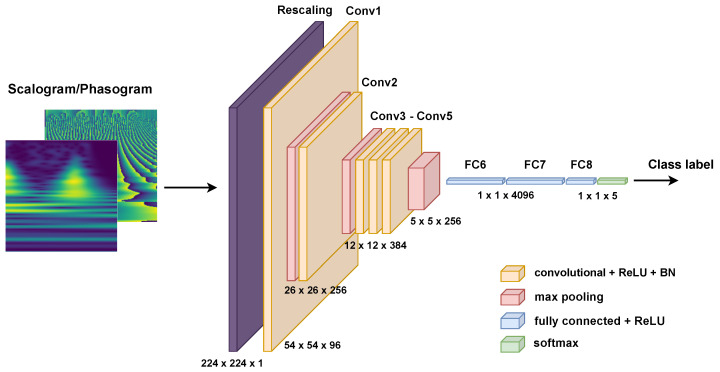
A sketch of the proposed customized AlexNet.

The methodology relies on a basic and simple network used for classifying a single input representation (i.e., only scalograms or phasograms), which is then modified according to the used data fusion strategy. The proposed basic architecture is based on a custom version of AlexNet [44] and used to evaluate the effectiveness of fusing both magnitude and phase information extracted by the CWT. This customized version of AlexNet is composed of five groups of convolutional layers, each followed by a batch normalization layer. The first, second, and fifth groups also have a MaxPooling layer for the reduction of the feature maps size. After a flattening operation, three dense (fully connected) layers are used, with the first two followed by a dropout operation with a drop rate of 0.5. The last layer use the softmax activation to obtain the classification outcome. A sketch of the proposed customized version of AlexNet is shown in Figure 5, while its organization and number of parameters are summarized in Table 2. For simplifying future reference to the architecture, we provide a name to each layer listed in the third column of Table 2. Moreover, to handle integer data, a rescaling layer was incorporated into the customized version of AlexNet. This layer transforms the integer input data back into the floating range [0,1]. Differently from the original version of AlexNet, the customized one works on a single channel input (i.e., the scalogram or the phasogram) and is fed by an image of size 224×224 (instead of 227×227).

The use of the AlexNet-based network is justified for the simplicity of its architecture in order to effectively evaluate the advantages of the different investigated fusion strategies. Since it is composed of a short sequence of convolutional layers and does not contain any shortcut connections, it is easier to evaluate the effect of the fusion of scalograms and phasograms. However, the proposed idea can be easily generalized to more complex networks (such as GoogLeNet, ResNet, and similar). In any case, some studies evaluating the performance of using scalograms of ECGs for biometric applications on AlexNet, GoogLeNet, and ResNet highlighted that these networks show similar performance on test accuracy (refer to Tables 2 and 4 of [32]).

### 3.5. Data Fusion

**Figure 6:**
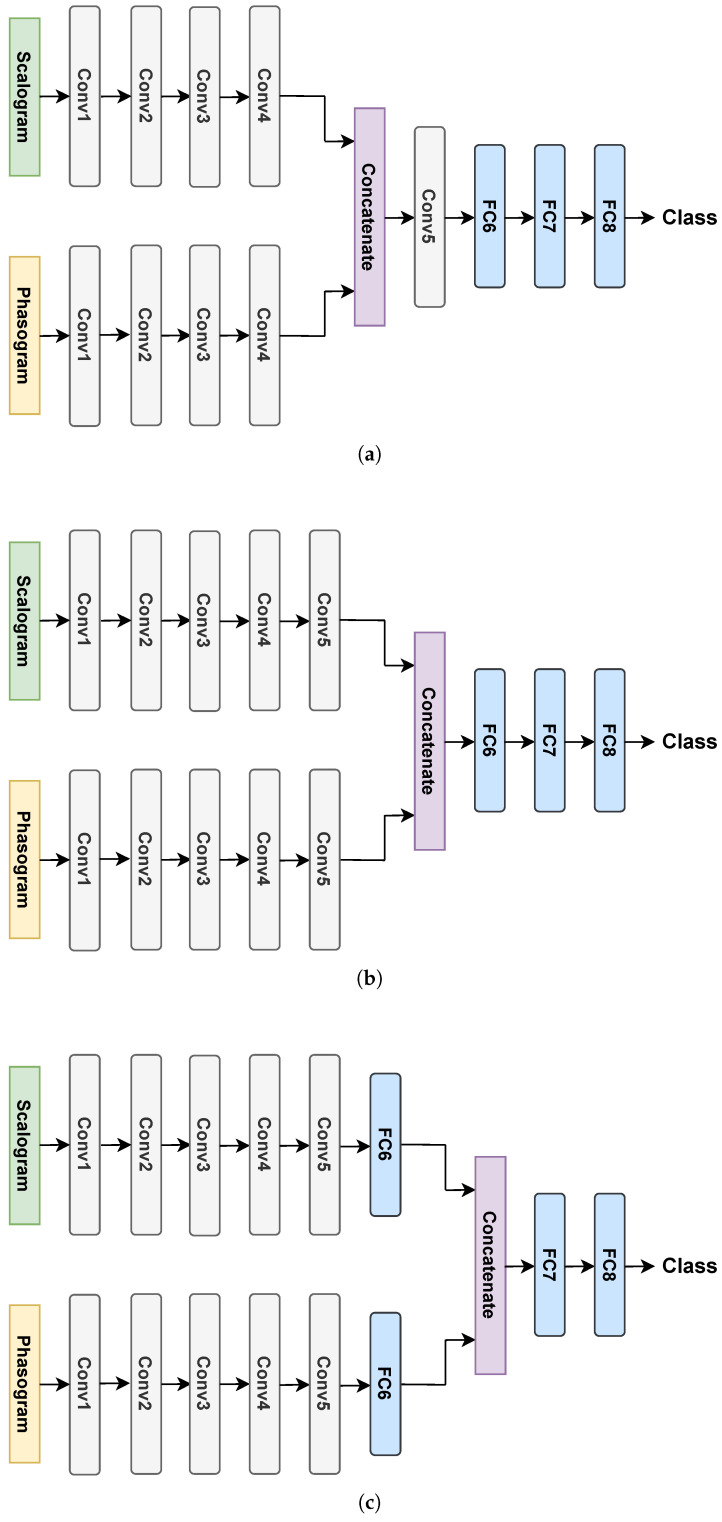
Schemes of the implemented S4, S5, and S6 fusion strategies. For simplicity, only the convolutional and dense layers are shown. For the layer names, refer to the third column of Table 2. (**a**) Fusion strategy S4. (**b**) Fusion strategy S5. (**c**) Fusion strategy S6.

In this work, we use the proposed AlexNet-based architecture introduced in Section 3.4 on scalograms and phasograms alone; then, we also consider different early, intermediate, and late fusion strategies. Specifically, a total of fourteen strategies are employed, denoted as follows:

1. S1: The strategy implementing the basic model of Table 2 and shown in Figure 5 fed with scalograms only.
2. S2: The strategy implementing the basic model of Table 2 and shown in Figure 5 fed with phasograms only.
3. S3: The strategy implementing the early fusion—both scalograms and phasograms are concatenated as an image input with two channels. In this case, the architecture is the same as in Table 2 and shown in Figure 5 but layers Input and Rescaling work with two channels instead of only one (224×224×2), i.e., the network is fed by both the scalogram and phasogram.
4. S4: The strategy implementing a first intermediate fusion idea, where the fusion is performed between the Conv4 and Conv5 convolutional layers. More specifically, two basic models working on scalograms and phasograms are composed of the first 12 layers in Table 2; then, after their concatenation, the flow continues from layer 13 to the end. The S4 model is sketched in Figure 6a (for simplicity and clarity, only the convolutional and dense layers are shown in the figure).
5. S5: The strategy implementing a second intermediate fusion idea, where fusion is performed before the first dense layer FC6. More specifically, two base models working on scalograms and phasograms are composed of the first 16 layers in Table 2; then, after their concatenation, the flow continues from layer 17 to the end. The S5 model is sketched in Figure 6b.
6. S6: The strategy implementing a third intermediate fusion idea, where fusion is performed between the FC6 and FC7 dense layers. More specifically, two base models working on scalograms and phasograms are composed of the first 17 layers in Table 2, without the dropout in layer Dr6; then, after their concatenation, the flow continues from layer 19 to the end. The S6 model is sketched in Figure 6c.
7. S7: Several late fusion strategies, in which we fuse the two predictions of models S1 and S2 in Figure 5 by using the following different algorithms:
  (a) S7a: Mean of the two inputs;
  (b) S7b: Linear SVM, with the regularization parameter set to C=1;
  (c) S7c: Decision tree;
  (d) S7d: Random forest, with 500 estimators;
  (e) S7e: Logistic regression, with the inverse of regularization strength set to C=1;
  (f) S7f: Naïve Bayes;
  (g) S7g: kNN, with k=11 neighbors;
  (h) S7h: Linear classifier, using an $\ell_{2}$ regularization with strength set to 0.5.

The computational complexity of implemented strategies, in terms of number of parameters, is summarized in Table 3. We can observe that the number of parameters increases from strategy S1 to strategy S6. In particular, intermediate fusions in S5 and S6 show the higher values. For the strategy S7, in addition to the parameters of strategies S1 and S2, we have to also consider the complexity of the specific chosen fusion algorithm.

### 3.6. Training and Hyper-Parameter Settings

All the considered models were trained by using the Adam optimizer, a variant of the gradient descent [105]. Specifically, the Adam algorithm includes estimates of the first- and second-order moments of the gradient, along with a bias correction, to accelerate the convergence process. Further details about the Adam algorithm are available in [105].

The Adam learning rate is set to $\eta =10^{-4}$, while the other parameters $\beta_{1}$, $\beta_{2}$, and ε are left at their default values. In this work, we use a batch size of B=32. The whole training procedure ran for 30 epochs, while the early stopping strategy, with five epochs of patience, was used.

Experiments were run on a standard office PC equipped with an Intel 14-th generation i9-14900KF CPU, 32 GB of RAM memory, and a Geforce RTX 3050 8 GB GPU. All the source code (Source code can be downloaded from https://github.com/mscarpiniti/ECG_Arrhythmia, accessed on 15 October 2024.) was implemented in Python by using the Anaconda environment.

### 3.7. Evaluation Metrics

The proposed strategies are evaluated by the confusion matrices that collect, for each class, the number of correctly and incorrectly classified entries. For each class, it is possible to identify the following:

- True Positive (TP): the number of samples belonging to a class that are correctly classified as the same class;
- False Positive (FP): the number of samples belonging to different classes that are incorrectly assigned to the actual class;
- True Negative (TN): the number of samples belonging to different classes that are correctly assigned;
- False Negative (FN): the number of samples belonging to the actual class that are incorrectly assigned to different classes.

These values are used to evaluate the proposed strategies by computing some well-known indices:

$$\mathrm{Precision}=\frac{\mathrm{TP}}{\mathrm{TP}+\mathrm{FP}},$$

which is the fraction of relevant beats among all the retrieved ones;

$$\mathrm{Sensitivity}=\frac{\mathrm{TP}}{\mathrm{TP}+\mathrm{FN}},$$

which is the fraction of relevant beats that have been retrieved;

$$\mathrm{Specificity}=\frac{\mathrm{TN}}{\mathrm{TN}+\mathrm{FP}},$$

which is the proportion of true negatives correctly identified;

$$F1-\mathrm{score}=2\;\frac{\mathrm{Precision}\times \mathrm{Sensitivity}}{\mathrm{Precision}+\mathrm{Sensitivity}},$$

which is the harmonic mean of Precision and Sensitivity.

After evaluating the previous indices for each class, we also compute the weighted averages of these indices with respect to the number of entries of each class to account for class imbalance. Moreover, we also use the overall Accuracy metric,

$$\mathrm{Accuracy}=\frac{\mathrm{TP}+\mathrm{TN}}{\mathrm{TP}+\mathrm{TN}+\mathrm{FP}+\mathrm{FN}},$$

which measures the fraction of correctly classified beats among the total number of beats in the test set. All these indices are expressed in percentage (%).

Finally, we evaluate the Matthews correlation coefficient (MCC):

$$\mathrm{MCC}=\frac{\mathrm{TP}\times \mathrm{TN}-\mathrm{FP}\times \mathrm{FN}}{\sqrt{\left(\mathrm{TP}+\mathrm{FP})(\mathrm{TP}+\mathrm{FN})(\mathrm{TN}+\mathrm{FP})(\mathrm{TN}+\mathrm{FN}\right)}},$$

which is of particular significance for strongly imbalanced datasets and assumes values inside the interval [−1,1]. A coefficient close to 1 represents a very good prediction, 0 an average random prediction, and −1 represents an inverse prediction.

## 4. Results and Discussion

### 4.1. Comparisons of Data Fusion Strategies

**Figure 7:**
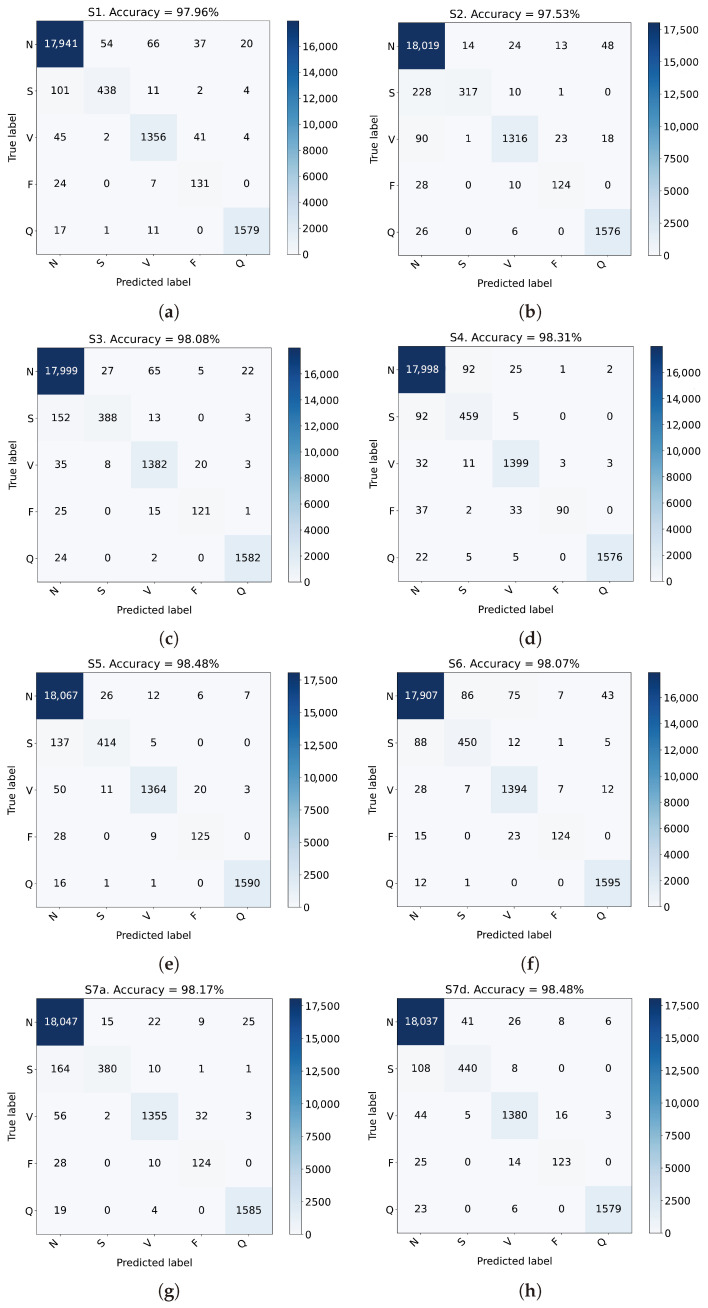
Confusion matrices of the considered strategies detailed in Table 4, Table 5, Table 6, Table 7, Table 8, Table 9, Table 10 and Table 11. (**a**) Confusion matrix of strategy S1. (**b**) Confusion matrix of strategy S2. (**c**) Confusion matrix of strategy S3. (**d**) Confusion matrix of strategy S4. (**e**) Confusion matrix of strategy S5. (**f**) Confusion matrix of strategy S6. (**g**) Confusion matrix of strategy S7a. (**h**) Confusion matrix of strategy S7d.

First, we evaluate the effectiveness of the proposed fusion strategies S1–S6 by training all of them on the training set and evaluating performance on the test set. Results for all considered strategies S1–S6 are summarized in Table 4, Table 5, Table 6, Table 7, Table 8 and Table 9, while a graphical representation of the related confusion matrices can be found in Figure 7.

An examination of results obtained by strategies S1 and S2, shown in Table 4 and Table 5, and Figure 7a,b, trained separately on scalograms and phasograms, respectively, highlights that strategy S1 performs slightly better than S2 by considering the weighted averages. This implies that scalograms are generally more discriminative with respect to the related phasograms. However, by a careful check of columns of Table 4 and Table 5, we argue that the sensitivity of class N is larger for strategy S2, as well as the precision of classes S, V, and F. Also, the F1-score obtained from strategy S2 for class F is greater than the corresponding one provided by strategy S1. With regard to the confusion matrices in Figure 7a,b, strategy S1 provides a larger number of true positive entries, except for class N, for which strategy S2 generates a larger number of true positive matches.

Although phasograms seem to be less discriminative than the corresponding scalograms, their joint use provides some advantages, and the results using both information show a general improvement, demonstrating the effectiveness of the idea to fuse both the magnitude and phase information. To this purpose, Table 6 and Figure 7c show the confusion matrix and considered metrics for the strategy S3, which implements the early data fusion, i.e., the input to the customized architecture described in Table 2, and Figure 5 has two channels composed of the scalogram (first channel) and the phasogram (second channel), respectively. Except for the specificity, all the other metrics are slightly improved by using the strategy S3, obtaining weighted averages over the 98%. Just the sensitivity of class S is smaller than the corresponding one obtained by strategy S1.

Results of the three considered intermediate fusion strategies implemented by models in S4, S5, and S6, respectively, are shown in Table 7, Table 8 and Table 9 and Figure 7d–f. Generally, all these implemented strategies outperform the early fusion one in S3, and the single S1 and S2 ones, highlighting the effectiveness of fusing input-specific features. However, the fusion before the second dense layer (strategy S6) does not provide great enhancements, since results shown in Table 9 are quite similar to those obtained by strategy S3 in Table 6. The best-performing strategy is the S5 one, i.e., the model where fusion is performed before the first dense layer. For this model, the overall accuracy is about 98.5%, with similar values for the weighted averages of F1-score, precision, and sensitivity; only the specificity is about 95%, lower than the other strategies. Strategy S4 behaves with an intermediate performance with respect to strategies S5 and S6.

Second, for the evaluation of the late fusion strategy S7, we used the previously trained models in S1 and S2 to generate the predictions produced by considering scalograms and phasograms alone, respectively, and then exploiting such predictions in several late fusion strategies, as detailed at the end of Section 3.5. Due to space limitation, in the following, we only show detailed results related to the mean of the two predictions (strategy S7a) and the use of a random forest classifier (strategy S7d). The corresponding numerical results are reported in Table 10 and Table 11 (and Figure 7g,h), respectively. However, most of the other late fusion strategies, except for the too simple Naïve Bayes, perform very similarly, as will be shown later in the comparative Table 12. From a careful examination of Table 10 and Table 11, and Figure 7g,h, we see that both the strategies produce an F1-score, precision, and recall higher than 98%. Interestingly enough, even a very simple strategy, like the mean in S7a, is able to produce good results (see Table 10), since average performance is only slighter lower than the corresponding S7d one, which behaves better in classifying classes S and V, as shown in Figure 7g,h. This is due to the fact that the two single predictions complement each other, since each model produces uncorrelated errors, once again highlighting the complementarity of the magnitude and phase information. The only remark is regarding the performance corresponding to the classes S and F, for which strategy S7a shows a lower F1-score with respect to the S7d one (mainly due to a lower sensitivity in class S and a lower precision in class F).

A comparison of all the implemented strategies in terms of overall accuracy, weighted F1-score, precision, sensitivity, and specificity is reported in Table 12. The table also shows results of all the considered late fusion strategies. Moreover, Table 12 also reports the Matthews correlation coefficient (MCC), which is of particular significance since the dataset is strongly imbalanced. From a careful examination of this table, we can argue that the proposed S5 and S7d (late fusion with random forest) strategies performs similarly: the only difference is in the specificity, which is slightly larger for the random forest late fusion. In addition, all the analyzed strategies, except once again the Naïve Bayes, behave very well against the data imbalance, as demonstrated by the values assumed by the MCCs that are close to 0.95 for the best strategies. As a concluding remark, except the Naïve Bayes and slightly the Decision Tree strategies, all the considered intermediate and late fusion strategies perform similarly, with the preference of the S5 and S7d strategies that show the best performance for all the considered metrics.

Overall, results shown in this sub-section demonstrate that the fusion of both the magnitude and phase information has a positive and effective impact on the classification performance.

### 4.2. Comparisons with the State of the Art

The proposed methodology is compared to other state-of-the-art approaches based on different methodologies but evaluated on the same dataset: the MIT-BIH Arrhythmia database. Specifically, we have compared the results obtained by strategy S5, which is resulted as the best performing, to the results of some of the works introduced in Section 2 that rely on the same dataset and the same number of classes, including some old works (like those in [9] or [49]) and other recent ones (such as [55,79]). Results of these comparisons with other state-of-the-art approaches are shown in Table 13.

This table confirms that, although a very simple CNN architecture was used (i.e., an AlexNet-based network), the proposed idea of simultaneously considering both the magnitude and phase information provides results that are competitive with respect to other state-of-the-art approaches. In fact, all considered metrics obtained by the proposed strategy S5 generally outperform those obtained by the other compared approaches, even if only by a small amount.

Specifically, oldest works exploiting ECG morphology, such as [9,49], present a lower accuracy and, in general, lower values for the other metrics, particularly for the approach in [49] that provides a sensitivity of just 78%. Also, the use of a CNN applied to the morphology shows an accuracy of about 93.2% and similar values for the other metrics [26]. Just when beats are analyzed at multiple scales as narrow-band Fourier intrinsic band functions after a Fourier decomposition, some traditional ML classifiers provide good results [55], similar to the proposed approach. Slightly better are the results obtained by using high-order statistics, such as [52,53,54], whose accuracies reach 94.5%. Similar results are obtained by using Random Forests [27], SVMs [51], DBNs [58,59], and their ensembles [90,91]. In these approaches, accuracy varies in the range 89.2% to 97.7%, lower than the proposed approach. In addition, for these methods, except the CraftNet in [90] that shows a poor F1-score, the other approaches provide similar precision, sensitivity, F1-score, and specificity values. A similar behavior was observed by using stacked autoencoders [57]. However, all the previous approaches show general performance lower than the proposed fusion ideas.

Regarding the use of 1D-CNNs, the results of the analyzed approaches span an accuracy from 88.3% of the simple approach in [69], which uses transfer learning, to 98.3% of the more complicated and recent approach in [79], where the 1D-CNN is followed by a recurrent network. All the other approaches based on the 1D-CNN behave in the middle between these two values. Generally, most of these approaches show a low sensitivity, except those involving a recurrent layer, whose sensitivities overcome 97%. However, using a recurrent layer after a fully connected network provides lower results [80]. In any case, we have to underline that the use of recurrent layers implies a large number of parameters and, hence, a computational cost higher than the proposed approach. The use of a U-Net does not provide better results, since the accuracy obtained in [63,64] is about 96%, with similar values for the other considered metrics. Better results are obtained by approaches exploiting 2D-CNNs that usually show accuracy of the order of 97%, such as in [28,77,87,88]. The results obtained by this family of approaches demonstrate the power of the bi-dimensional representation of the ECG signals. Although approaches based on 2D-CNNs show very good results, all approaches analyzed in Table 13 provide slightly lower performance than the proposed one. Just the approach proposed in [25] shows an accuracy larger than the proposed one; however, such an approach provides lower F1-score, precision, and sensitivity metrics, which are very important and effective metrics in medical scenarios. As an additional remark, we point out that the approach introduced in [62] behaves very similar to the proposed one, since all metrics (except the sensitivity) are slighter smaller, while the sensitivity is slightly larger.

Overall, the fusion of scalograms and phasograms allows a simple architecture like AlexNet, composed of a small number of convolutional layers, to compete with many state-of-the-art approaches. The simple AlexNet, which has a small number of parameters compared to more complex solutions, also has the advantage of a simple investigation of the feature maps to understand which time-scale regions of the extracted features are responsible for the final decision (see Section 4.4). Hence, we can conclude that the proposed approach is an effective method for the detection of arrhythmia and is able to compete with most of the state-of-the-art approaches.

### 4.3. Cross-Dataset Independent Validation

We also performed an independent validation by testing our models trained on the MIT-BIH database on a second dataset: the St. Petersburg INCART 12-lead Arrhythmia Database (It can be downloaded at https://physionet.org/content/incartdb/1.0.0/, accessed on 2 December 2024). This dataset, which is part of the PhysioNet/Computing in Cardiology Challenge 2020 and 2021 (See https://moody-challenge.physionet.org/, accessed on 2 December 2024), consists of 75 half-hour 12-lead annotated recordings extracted from 32 Holter records of both male and female patients. It is composed of more than 175,000 heartbeats (most in classes N, V, and S, while classes F and Q have only 215 and 6 beats, respectively) recorded at 257 Hz as sampling rate. This dataset has also been used in literature for arrhythmia detection using deep learning models [106,107].

From this dataset, we have selected a subset composed of 15,000 heartbeats of class N, 1000 beats of class S, 2000 of class V, 215 of class F, and 6 of class Q. Scalograms and phasograms of this subset were extracted by using the same preprocessing procedure described in Section 3.2 after selecting only the lead II (the same used in MIT-BIH). These new scalograms and phasograms are then used as a new test set for the proposed models. Specifically, we evaluate the performance for all strategies S1–S6, while we select strategies S7a and S7d for late fusion, since the other ones perform similarly.

Results of the implemented models on the independent dataset, containing subjects unseen during the training phase, are shown in Table 14. Although the performance on this independent dataset is lower compared to results obtained with the MIT-BIH database (shown in Table 12), metric values reported in Table 14 confirm the general considerations discussed in Section 4.1. The fusion of both the magnitude and phase information (in strategies S3–S6, S7a, and S7d) provides larger values for all the metrics with respect to the use of the single information (in strategies S1 and S2). In addition, the improvement in performance, in this case, is larger with respect to the one observed in Table 12 when the MIT-BIH database was used. Once again, the best strategy is the intermediate fusion S5 followed by the late fusion S7d; generally, the global performance hierarchy is conserved.

### 4.4. Interpretability

**Figure 8:**
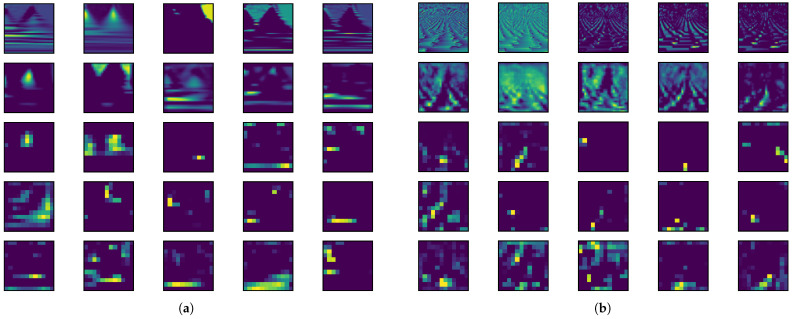
Five randomly selected feature maps for each layer related to (**a**) scalograms and (**b**) phasograms. The row is related to the network layer.

We note that scalograms and phasograms effectively capture significant localized time-scale events in ECG signals, evident from the horizontal lines or cloud-like points in Figure 4. Additionally, the convolutional layers of the CNN can learn distinctive features, as demonstrated in Figure 8, which presents five randomly selected feature maps from each convolutional layer for both the scalogram and phasogram branches using the S5 architecture. Although each channel encodes relatively independent features, due to the huge number of feature maps in the whole architecture and the space constraints of the paper, we randomly selected only five feature maps for each convolutional layer. Even if the single plots in Figure 8 are quite small, it is clear that feature maps in the final layer are more specialized in detecting a specific time scale: in fact, scalograms in the last row of Figure 8a show clear horizontal lines localized at a specific scale. In fact, generally, feature maps become more abstract and specialized by passing from the first layer (first row in Figure 8), where filters act as edge detectors, to following layers until the fifth one (fifth row in Figure 8), where maps are more abstract, sparse, and less interpretable. A similar reasoning can be generalized to phasograms in Figure 8b. Hence, taking into account both scalograms and phasograms simultaneously, the proposed architectures can exploit features that are very localized in both time and scale domains. This behavior justifies the better performance of the proposed approach for the classification of arrhythmia in ECGs.

**Figure 9:**
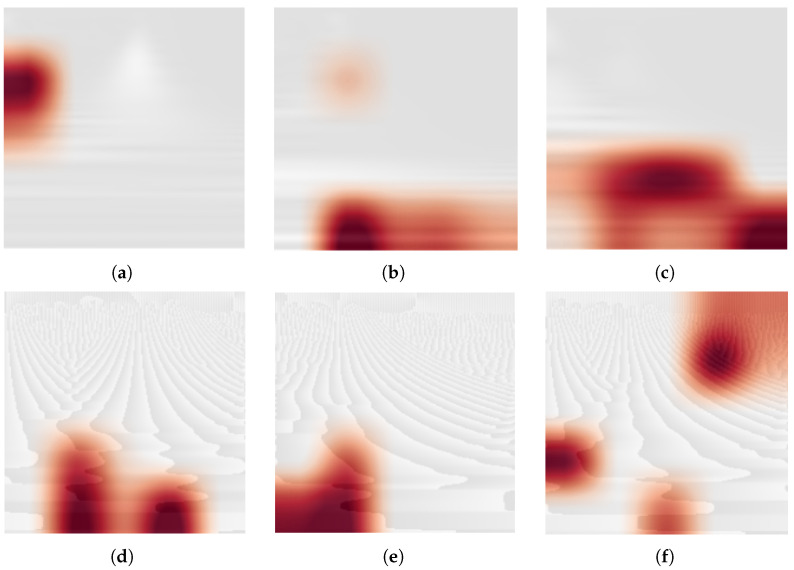
Gradient-weighted Class Activation Mapping (Grad-CAM) [108] of the last convolutional layer related for some scalograms (top row) and related phasograms (bottom row) corresponding to three instances extracted from classes N, F, and V, respectively. (**a**) Class N. (**b**) Class V. (**c**) Class F. (**d**) Class N. (**e**) Class V. (**f**) Class F.

Moreover, Figure 9 shows the Gradient-weighted Class Activation Mapping (Grad-CAM) [108] of the last convolutional layer for some randomly selected scalograms and phasograms related to the N, F, and V classes and using the strategy S5. This visualization technique is useful for understanding which parts of an input image are responsible for the final classification decision. The general technique consists in producing heatmaps of the class activation over input images. Specifically, the gradient of the class with respect to each channel is used as a weight for every channel in a feature map generated by an input image. The produced heatmap is then superimposed to the input image. The images in Figure 9 were rendered by using a red color map over a gray-scale version of the input, while the gradient information appear as a red shadow. The figure shows that the trained network is able to selectively respond to specific input, since only small regions of the input images are responsible for the classification decision.

Finally, we investigate the effectiveness of the proposed fusion strategies by resorting to the SHAP (SHapley Additive exPlanations) approach, a model-agnostic method used to explain the output of a machine learning model [109,110], particularly effective for image classification [111]. Specifically, the SHAP explanation method, derived from the game theory, computes Shapley values and aims at explaining the prediction of an instance by computing the contribution of each feature to that prediction. For the image classification problem, the basic goal is to assess the contribution of each pixel to a specific class. For this purpose, SHAP values are calculated from a trained network and visualized as heatmaps, by highlighting pixels with a positive contribution to the classification of the actual class in red color and pixels with a negative contribution in blue color.

**Figure 10:**
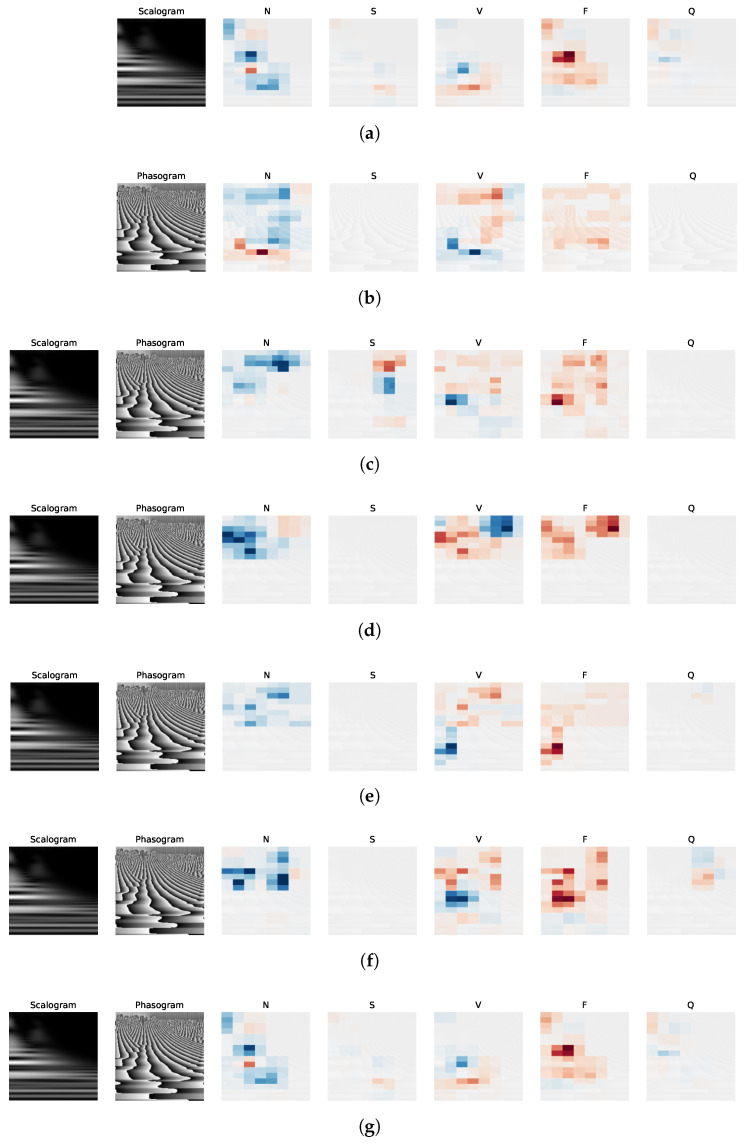
SHAP values obtained by the implemented strategies S1–S6 and S7d by using an input belonging to class F. (**a**) S1. (**b**) S2. (**c**) S3. (**d**) S4. (**e**) S5. (**f**) S6. (**g**) S7d.

Figure 10 shows the Shapley values obtained by the implemented strategies S1–S6 and S7d (the best-performing late fusion strategy) by using an input belonging to class F. Figure 10a,b are related to the predictions of using only scalograms and phasograms, respectively. Heatmaps shown in the last five columns of the maps in Figure 10c–g (for strategies S3–S6 and S7d) are obtained by superimposing both the modalities and related SHAP values. From an analysis of this figure, we can recognize that all the proposed strategies were able to correctly classify the input instance as belonging to the actual class F, since many pixels of this class provide a positive contribution. In addition, by comparing heatmaps of Figure 10c–g with the corresponding one of Figure 10a,b, we can infer that the strategies adopting the data fusion (S3–S6 and S7d) exploit both the modalities, since the related heatmaps incorporate positive contributions from both the scalograms and phasograms alone (compared to Figure 10a,b). Moreover, heatmaps of strategies S5 (in Figure 10e) and S7d (in Figure 10g) provide sparser maps, meaning that these approaches are able to take the decision by focusing only on a very specific region of the input scalograms and phasograms. Similar conclusions can be drawn by analyzing inputs belonging to the other classes. Overall, Figure 10 on the SHAP method supports the conclusion that the proposed intermediate and late fusion strategies, particularly S5 and S7d, benefit from both modalities.

## 5. Conclusions

In this paper, we investigated different data fusion strategies exploiting both scalograms and phasograms of ECG signals to detect arrhythmia diseases. Scalograms and phasograms are the squared magnitude and the phase of the continuous wavelet transform (CWT), respectively. The complex Morlet wavelet was used in this work. The investigated data fusion strategies rely on deep learning architectures based on convolutional neural networks (CNNs). The use of both the magnitude and phase information has some advantages over the use of the magnitude alone, and the proposed approach was able to compete with other state-of-the-art approaches. Some numerical results have shown the effectiveness of the proposed approach, by obtaining an overall accuracy of about 98.5%, and sensitivity and specificity of 98.5% and 95.6%, respectively.

Future researches will be focused toward the development of “phase-aware” models for the analysis of ECG signals. More specifically, future works will be addressed to examine other and more sophisticated strategies, such as autoencoder (AE)-based architectures and models exploiting the attention mechanism focused on the phase information.

## Figures and Tables

**Table 1:** Training and test splits for each class.

Class	Meaning	Proportion (%)	Samples	Training Samples	Test Samples
N	Normal beat	82.77	90,589	72,471	18,118
S	Supraventricular premature or ectopic beat	2.54	2779	2223	556
V	Premature ventricular contraction	6.61	7236	5788	1448
F	Fusion of ventricular and normal beat	0.73	803	641	162
Q	Unclassifiable beat	7.35	8039	6431	1608
Total		100.00	109,446	87,554	21,892

**Table 2:** Organization and number of parameters of the customized AlexNet.

Number	Layer Type	Layer Name	Output Shape	Number of Params
1	Input	Input	(None, 224, 224, 1)	0
2	Rescaling	Rescaling	(None, 224, 224, 1)	0
3	Conv2D	Conv1	(None, 54, 54, 96)	11,712
4	BatchNormalization	BN1	(None, 54, 54, 96)	384
5	MaxPooling2D	Pool1	(None, 26, 26, 96)	0
6	Conv2D	Conv2	(None, 26, 26, 256)	614,656
7	BatchNormalization	BN2	(None, 26, 26, 256)	1024
8	MaxPooling2D	Pool2	(None, 12, 12, 256)	0
9	Conv2D	Conv3	(None, 12, 12, 384)	885,120
10	BatchNormalization	BN3	(None, 12, 12, 384)	1536
11	Conv2D	Conv4	(None, 12, 12, 384)	1,327,488
12	BatchNormalization	BN4	(None, 12, 12, 384)	1536
13	Conv2D	Conv5	(None, 12, 12, 256)	884,992
14	BatchNormalization	BN5	(None, 12, 12, 256)	1024
15	MaxPooling2D	Pool5	(None, 5, 5, 256)	0
16	Flatten	Flatten	(None, 6400)	0
17	Dense	FC6	(None, 4096)	26,218,496
18	Dropout	Dr6	(None, 4096)	0
19	Dense	FC7	(None, 4096)	16,781,312
20	Dropout	Dr7	(None, 4096)	0
21	Dense	FC8	(None, 5)	20,485
			Total params:	46,749,765
			Trainable params:	46,747,013
			Non-trainable params:	2752

**Table 3:** Number of parameters of the considered strategies.

Strategy	Number of Parameters
S1	46,749,765
S2	46,749,765
S3	46,761,381
S4	50,477,957
S5	76,693,637
S6	93,474,949

**Table 4:** Confusion matrix and considered metrics for strategy S1.

		Predicted					F1-Score	Precision	Sensitivity	Specificity
		N	S	V	F	Q				
Original	N	17,941	54	66	37	20	98.99	98.97	99.02	95.05
	S	101	438	11	2	4	83.35	88.48	78.78	99.73
	V	45	2	1356	41	4	96.59	93.45	93.65	99.54
	F	24	0	7	131	0	70.24	62.09	80.86	99.63
	Q	17	1	11	0	1579	98.23	98.26	98.20	99.86
All classes							97.99	98.01	97.96	95.85

**Table 5:** Confusion matrix and considered metrics for strategy S2.

		Predicted					F1-Score	Precision	Sensitivity	Specificity
		N	S	V	F	Q				
Original	N	18,019	14	24	13	48	98.71	97.98	99.45	90.14
	S	228	317	10	1	0	71.40	95.48	57.01	99.93
	V	90	1	1316	23	18	93.53	96.34	90.88	99.76
	F	28	0	10	124	0	76.78	77.02	76.54	99.83
	Q	26	0	6	0	1576	96.98	95.98	98.01	99.67
All classes							97.52	97.50	97.53	91.80

**Table 6:** Confusion matrix and considered metrics for strategy S3.

		Predicted					F1-Score	Precision	Sensitivity	Specificity
		N	S	V	F	Q				
Original	N	17,999	27	65	5	22	99.02	98.71	99.34	93.75
	S	152	388	13	0	3	79.26	91.73	69.78	99.84
	V	35	8	1382	20	3	94.50	93.57	95.44	99.54
	F	25	0	15	121	1	78.57	82.88	74.69	99.88
	Q	24	0	2	0	1582	98.29	98.20	98.38	99.86
All classes							98.06	98.03	98.08	94.78

**Table 7:** Confusion matrix and considered metrics for strategy S4.

		Predicted					F1-Score	Precision	Sensitivity	Specificity
		N	S	V	F	Q				
Original	N	17,998	92	25	1	2	99.17	98.99	99.34	95.15
	S	92	459	5	0	0	81.60	80.68	82.55	99.48
	V	32	11	1399	3	3	95.99	95.36	96.62	99.67
	F	37	2	33	90	0	70.31	95.74	55.56	99.98
	Q	22	5	5	0	1576	98.84	99.68	98.01	99.98
All classes							98.31	98.31	98.31	95.95

**Table 8:** Confusion matrix and considered metrics for strategy S5.

		Predicted					F1-Score	Precision	Sensitivity	Specificity
		N	S	V	F	Q				
Original	N	18,067	26	12	6	7	99.23	98.74	99.72	93.88
	S	137	414	5	0	0	82.14	91.59	74.46	99.82
	V	50	11	1364	20	3	96.09	98.06	94.20	99.87
	F	28	0	9	125	0	79.87	82.78	77.16	99.88
	Q	16	1	1	0	1590	99.13	99.37	98.88	99.95
All classes							98.46	98.44	98.48	94.92

**Table 9:** Confusion matrix and considered metrics for strategy S6.

		Predicted					F1-Score	Precision	Sensitivity	Specificity
		N	S	V	F	Q				
Original	N	17,907	86	75	7	43	99.02	99.21	98.84	96.21
	S	88	450	12	1	5	81.82	82.72	80.94	99.56
	V	28	7	1394	7	12	94.44	92.69	96.27	99.46
	F	15	0	23	124	0	82.39	89.21	76.54	99.93
	Q	12	1	0	0	1595	97.76	96.37	99.19	99.70
All classes							98.07	98.08	98.07	96.80

**Table 10:** Confusion matrix and considered metrics for strategy S7a.

		Predicted					F1-Score	Precision	Sensitivity	Specificity
		N	S	V	F	Q				
Original	N	18,047	15	22	9	25	99.07	98.54	99.61	92.93
	S	164	380	10	1	1	79.75	95.72	68.35	99.92
	V	56	2	1355	32	3	95.12	96.72	93.58	99.77
	F	28	0	10	124	0	75.61	74.70	76.54	99.80
	Q	19	0	4	0	1585	98.39	98.20	98.57	99.86
All classes							98.16	98.15	98.17	94.12

**Table 11:** Confusion matrix and considered metrics for strategy S7d.

		Predicted					F1-Score	Precision	Sensitivity	Specificity
		N	S	V	F	Q				
Original	N	18,037	41	26	8	6	99.23	98.90	99.55	94.70
	S	108	440	8	0	0	84.45	90.53	79.14	99.78
	V	44	5	1380	16	3	95.77	96.23	95.30	99.74
	F	25	0	14	123	0	79.61	83.67	75.93	99.89
	Q	23	0	6	0	1579	98.81	99.43	98.20	99.96
All classes							98.46	98.44	98.48	95.59

**Table 12:** Summary and comparisons of the implemented models in terms of the considered metrics (all reported in percentage (%) except the MCC).

Fusion	Strategy	Accuracy	F1-Score	Precision	Sensitivity	Specificity	MCC
Single	S1	97.96	97.99	98.01	97.96	95.85	0.9329
	S2	97.53	97.52	97.50	97.53	91.80	0.9173
Early	S3	98.08	98.06	98.03	98.08	94.78	0.9363
	S4	98.31	98.31	98.31	98.31	95.95	0.9441
Intermediate	S5	98.48	98.46	98.44	98.48	94.92	0.9495
	S6	98.07	98.07	98.08	98.07	96.80	0.9372
Late	S7a	98.17	98.16	98.15	98.17	94.12	0.9389
	S7b	98.40	98.38	98.36	98.40	95.30	0.9467
	S7c	97.97	97.96	97.96	97.97	95.27	0.9330
	S7d	98.48	98.46	98.44	98.48	95.59	0.9495
	S7e	98.42	98.40	98.38	98.42	95.37	0.9475
	S7f	95.93	96.69	97.46	95.93	97.85	0.8782
	S7g	98.39	98.37	98.35	98.39	95.28	0.9464
	S7h	98.37	98.36	98.34	98.37	95.04	0.9458

**Table 13:** Comparisons with other state-of-the-art approaches using the MIT-BIH Arrhythmia database, listed in temporal order (from the oldest to the most recent).

Ref.	Approach	Accuracy	F1-Score	Precision	Sensitivity	Specificity
[9]	ECG morphology	85.88	90.23	95.06	85.88	94.35
[49]	ECG morphology	78.00	86.00	95.83	78.00	96.70
[52]	HOS	94.52	98.98	99.36	98.61	98.41
[53]	HOS + SVM	92.77	98.67	99.30	98.04	98.26
[58]	DBN	96.10	96.11	96.11	96.10	98.96
[27]	Random Forests	94.61	93.04	94.62	91.52	96.32
[71]	1D-CNN	92.80	92.83	92.86	92.80	79.26
[61]	1D-CNN	93.47	96.93	97.87	96.01	91.64
[57]	Stacked AE	97.49	97.54	97.59	97.49	88.72
[69]	1D-CNN + TL	88.30	90.67	93.17	88.30	92.75
[59]	DBN	93.78	48.72	33.63	88.39	93.32
[75]	1D-CNN + LSTM	97.88	97.87	98.48	97.26	98.50
[51]	SVM	97.78	97.74	97.72	97.78	90.98
[83]	2D-CNN + DF	96.05	96.17	96.30	96.05	93.12
[78]	1D-CNN + RNN	97.80	97.34	95.76	98.98	96.95
[82]	2D-CNN + WT	92.96	92.86	92.81	92.96	07.73
[91]	ML Ensemble	94.47	94.90	95.34	94.47	87.72
[63]	U-Net	96.13	96.09	96.05	96.13	95.84
[26]	Morphology + CNN	93.19	94.60	94.20	95.00	93.98
[92]	1D-CNN + BiLSTM	96.77	77.84	81.24	74.89	95.16
[20]	1D-CNN + LSTM	96.62	95.48	95.56	95.40	96.80
[54]	HOS + Markov Models	88.33	88.37	88.40	88.33	94.17
[90]	CraftNet	89.24	73.06	61.84	89.25	95.79
[73]	DNN + 1D-CNN	95.69	96.17	96.66	95.69	85.51
[70]	2D-CNN + TL	93.52	93.66	93.12	94.21	93.26
[80]	DenseNet + BiLSTM	92.37	63.49	60.35	68.29	94.51
[17]	ResNet-18	96.49	96.57	96.66	96.49	97.48
[88]	CWT + 2D-CNN	97.33	97.37	97.41	97.33	98.73
[77]	2D-CNN + DF	97.59	97.30	97.47	97.14	97.95
[76]	1D-CNN + Attention	96.60	96.76	96.91	96.60	99.15
[87]	CWT + 2D-CNN	97.48	97.13	96.78	97.48	86.56
[97]	1D-CNN + ResNet	95.00	86.50	87.00	86.00	97.00
[64]	U-Net	95.55	95.53	95.52	95.55	97.64
[65]	1D-CNN + LSTM	97.58	97.56	97.54	97.58	90.50
[25]	1D-CNN + ELM	98.82	93.52	93.90	93.14	94.73
[28]	CWT + 2D-CNN + SVM	97.22	97.22	97.23	97.22	98.61
[62]	1D-CNN	98.41	98.40	98.40	98.41	96.07
[67]	1D-CNN + Transformer	97.66	93.99	93.83	94.16	74.00
[79]	1D-CNN + LSTM	98.24	92.07	98.92	86.10	97.50
[55]	Morphology + ML	98.01	97.66	97.31	98.01	92.08
Proposed (S5)	CWT + 2D-CNN + DF	98.48	98.46	98.44	98.48	95.59

**Table 14:** Performance of the implemented models on the independent St. Petersburg INCART Arrhythmia Database in terms of the considered metrics (all reported in percentage (%) except the MCC).

Fusion	Strategy	Accuracy	F1-Score	Precision	Sensitivity	Specificity	MCC
Single	S1	90.53	91.30	92.09	90.53	90.55	0.7157
	S2	89.86	90.75	91.66	89.86	90.18	0.6982
Early	S3	91.18	91.75	92.32	91.18	90.55	0.7313
	S4	91.28	91.91	92.56	91.28	91.47	0.7358
Intermediate	S5	91.93	92.42	92.92	91.93	91.63	0.7526
	S6	91.28	91.86	92.45	91.28	91.06	0.7350
Late	S7a	91.40	91.99	92.59	91.40	91.26	0.7379
	S7d	91.91	92.40	92.89	91.91	91.63	0.7524

## Data Availability

The data presented in this study can be downloaded from the PhysioNet portal at https://www.physionet.org/content/mitdb/1.0.0/ (accessed on 16 September 2024) and https://physionet.org/content/incartdb/1.0.0/ (accessed on 2 December 2024).

## Abbreviations

The following abbreviations are used in this manuscript:

AAMI	American Association of Medical Instrumentation
AE	Autoencoder
AI	Artificial Intelligence
CAM	Class Activation Mapping
CNN	Convolutional Neural Network
CWT	Continuous Wavelet Transform
DBN	Deep Belief Network
DF	Data Fusion
DNN	Deep Neural Network
DL	Deep Learning
DRNN	Deep Recurrent Neural Network
ECG	Electrocardiogram
ELM	Extreme Learning Machine
GAF	Gramian Angular Field
GRU	Gated Recurrent Unit
HOS	Higher-Order Statistic
LSTM	Long Short-Term Memory
ML	Machine Learning
MLP	Multilayer Perceptron
NN	Neural Network
RNN	Recurrent Neural Network
STFT	Short-Time Fourier Transform
SVM	Support Vector Machine
TL	Transfer Learning
WT	Wavelet Transform
